# A PORTABLE TECHNOLOGY-BASED BALANCE ASSESSMENT PREDICTS FALL RISKS IN LOW-INCOME COMMUNITY-DWELLING OLDER ADULTS

**DOI:** 10.1093/geroni/igad104.3145

**Published:** 2023-12-21

**Authors:** Evette Trahan, Renata Komalasari, Ladda Thiamwong

**Affiliations:** University of Central Florida, Orlando, Florida, United States; Penn State Nursing, State College, Pennsylvania, United States; University of Central Florida, Orlando, Florida, United States

## Abstract

Despite strong evidence on the association between balance performance and fall risks, there is a need for further studies among racially diverse low-income communities. Sixty-two healthy, low-income community-dwelling older adults (mean 74.11, SD: 6.34, 61-88 years old) in Central Florida were recruited and administered the BTrackS Balance Tracking System (BBS) for measuring balance performance. BBS is a postural sway-based balance performance assessment, where participants were guided to stand still on the BBS plate with hands on hips and eyes closed for about 1-2 minutes. Fall risk was assessed by the Centers for Disease Control and Prevention (CDC)’s twelve-item self-assessment checklist, Stopping Elderly Accidents, Deaths & Injuries (STEADI). About 58% of participants were African Americans, completed high school (54.8%), lived alone (67.7%), and earned just enough financially (54.8%). The simple regression results indicated that the model explained 14.9% of the variance (R2=.149, F (1,60) = 10.47, p =.002). Higher BBS scores (β = .100, p =.002) were significantly associated with higher fall risk scores. As older adults had reduced balance, their fall risks increased. The findings of this study confirmed the benefits of using a combination of a simple fall risk checklist and a portable assessment technology in enhancing the accessibility of balance and fall risk screening. Furthermore, a technology-based balance performance assessment is key in tailoring fall interventions for older adults with poor balance.

